# Origin of micro-scale heterogeneity in polymerisation of photo-activated resin composites

**DOI:** 10.1038/s41467-020-15669-z

**Published:** 2020-04-15

**Authors:** Slobodan Sirovica, Johanne H. Solheim, Maximilian W. A. Skoda, Carol J. Hirschmugl, Eric C. Mattson, Ebrahim Aboualizadeh, Yilan Guo, Xiaohui Chen, Achim Kohler, Dan L. Romanyk, Scott M. Rosendahl, Suzanne Morsch, Richard A. Martin, Owen Addison

**Affiliations:** 1 Faculty of Dentistry, Oral & Craniofacial Sciences, King’s College London, Guy’s Hospital, London, SE1 9RT UK; 2 Aston Institute of Materials Research, School of Engineering & Applied Science, Aston University, Birmingham, B4 7ET UK; 3 Faculty of Science and Technology, Norwegian University of Life Sciences, Ås, 1432 Norway; 4 ISIS Pulsed Neutron and Muon Source, Science and Technology Facilities Council, Rutherford Appleton Laboratory, Harwell Science and Innovation Campus, Didcot, OX11 0QX UK; 5 Department of Physics, University of Wisconsin-Milwaukee, Milwaukee, WI 53211 USA; 6 Department of Materials Science and Engineering, University of Texas at Dallas, 800 West Campbell Road, Richardson, TX 75080 USA; 7 Faculty of Medicine and Dentistry, University of Alberta, Edmonton, AB T6G 1C9 Canada; 8 Division of Dentistry, School of Medical Sciences, The University of Manchester, Manchester, M13 9PL UK; 9 Department of Mechanical Engineering, University of Alberta, Edmonton, AB T6G 1H9 Canada; 10 Canadian Light Source Inc., 44 Innovation Blvd., Saskatoon, SK S7N 2V3 Canada; 11 Corrosion and Protection Centre, School of Materials, The University of Manchester, Manchester, M13 9PL UK

**Keywords:** Polymers, Polymer characterization, Composites

## Abstract

Photo-activated resin composites are widely used in industry and medicine. Despite extensive chemical characterisation, the micro-scale pattern of resin matrix reactive group conversion between filler particles is not fully understood. Using an advanced synchrotron-based wide-field IR imaging system and state-of-the-art Mie scattering corrections, we observe how the presence of monodispersed silica filler particles in a methacrylate based resin reduces local conversion and chemical bond strain in the polymer phase. Here we show that heterogeneity originates from a lower converted and reduced bond strain boundary layer encapsulating each particle, whilst at larger inter-particulate distances light attenuation and monomer mobility predominantly influence conversion. Increased conversion corresponds to greater bond strain, however, strain generation appears sensitive to differences in conversion rate and implies subtle distinctions in the final polymer structure. We expect these findings to inform current predictive models of mechanical behaviour in polymer-composite materials, particularly at the resin-filler interface.

## Abstract (web summary)

Resin-based-composites are widely used in industry and medicine but the influence of the filler particles on the reactive group conversion in photocurable resins is yet to be elucidated. Here the authors observe reduced local conversion and chemical bond strain in silica filler acrylate composite using synchrotron-based wide-field IR imaging.

## Introduction

Photo-activated (meth) acrylate composites are of great interest for numerous applications, including composites for adhesives and coatings^1,2^, 3D printing^3–5^ and fabrication of aerospace^6^ and biomedical materials^7–10^. These composites combine a resin matrix with an inorganic filler phase and can be demand-set using light to excite a photo-initiator species dispersed within the matrix to initiate free radical polymerisation. The capability to set these composites in situ has driven their widespread use as a biomedical material, permitting clinical operators greater time to optimise the placement, sculpting and setting of the restorative in comparison with alternative materials. Historically, the development of biomedical resin composites has been driven by dental applications and consequently much of the research into photo-polymerised composites has been performed within this context.

Contemporary dental composites are based on dimethacrylate chemistry and typically achieve between 55 and 75% reactive group conversion under clinical setting parameters^9–11^. Following the initiation of free radical polymerisation, the system will experience an auto-acceleration of the conversion rate, reaching a rate maximum as the polymer forms cross-links between neighbouring chains. Composites with increased reactive group conversion can demonstrate superior strength, surface hardness, flexural and elastic modulus^12,13^ and limit the amount of residual unreacted monomer that may leach from the composite into surrounding tissues^14^. Nano and micron-scale inorganic filler particles are combined with the resin matrix to improve composite strength, toughness and wear resistance^9,10,15,16^. It has been reported that the addition of filler particles diminishes the generation of shrinkage stresses associated with volumetric shrinkage on curing^16,17^, at the expense of reactive group conversion^18^. Fourier transform infrared (FTIR) spectroscopy has been widely used for quantifying the degree of conversion (DC) of these materials^19–22^ and inhomogeneity in conversion has been detected at the macro-scale both laterally and through the material thickness^23^. The presence of filler particles introduces additional refractive indexes, light scattering and extinction coefficients, which differ to that of the dynamic properties of the resin matrix^24^. Monte Carlo simulations based on radiative transfer models have been applied to these systems to approximate light irradiance as a function of depth to extrapolate DC and matrix hardness values^25^. Simulation results correspond well with experimental data, demonstrating reduced light transmission, DC and hardness at increasing sample thickness relative to an unfilled (neat) resin matrix. To date, numerical solutions have not been extended to consider spatial variations in conversion at inter-particulate length scales, while experimental efforts have been hindered by technological limitations.

FTIR spectroscopy remains the most promising approach to determine the relationship between polymerisation variables and reactive group conversion. Mid-IR spectroscopy can be used to quantify DC in methacrylate systems by comparing aliphatic absorption bands in the monomer and polymer states, applied as either surface measurements (attenuated total reflection, ATR) or as transmission measurements on samples with a thickness <30 µm^22^. However, the key limitation of these studies is the lack of lateral resolution required to study regional reactive group conversion spatially at length scales relevant to variability in the material micro-structure. Consequently, the composite micro-structure with respect to the pattern of conversion at inter-particulate distances is unknown. This is important as the micro-structure, particularly at the interface between the resin and filler phase, will dictate final physico-mechanical properties and ultimately material performance.

Conventional FTIR micro-spectroscopic systems that perform in a dual-aperture confocal setting and equipped with a thermal source (e.g. globar) are limited by a trade-off between spatial resolution, which is diffraction limited, acquisition time, and signal-to-noise ratio (SNR). Coupling high throughput and SNR synchrotron beams with wide-field high-resolution imaging and oversampling permits faster data acquisition and an increase in SNR and spatial resolution compared with single beam sources^26^. The IRENI (InfraRed ENvironmental Imaging) beamline, an advanced synchrotron-based wide-field IR imaging system combining an IR microscope equipped with a multi-beam synchrotron source and a focal plane array (FPA) detector, allows spatial oversampling to overcome diffraction limitations and provide spatially-resolved chemical images for the entire mid-IR wavelength range (2.5–10 μm)^27^.

Localised variation in the photo-polymerisation of the interstitial resin matrix is yet to be elucidated but is essential for understanding structural variation in and the optimisation of photo-activated composite materials. Here, we apply the unique IRENI instrument and advanced Mie scattering corrections developed for this experimental system to a highly monodisperse model composite to allow the quantification of inter-particulate heterogeneity of conversion and associated residual stress states as a function of polymerisation variables in photo-polymerised composite materials. Synchrotron-based wide-field IR imaging combined with atomic force microscopy IR spectroscopy reveals reduced reactive group conversion and residual strains in the resin matrix within the immediate vicinity of filler particles. Results indicate the presence of a lower converted boundary layer, storing negligible strain compared with the inter-particulate resin matrix, encapsulating each filler particle.

## Results

### Local and inter-particulate structural heterogeneity

**Fig. 1:**
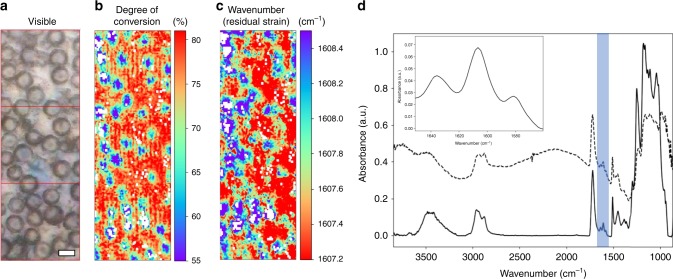
Visualisation of inter-particulate conversion and residual strain in polymer composites. **a** Bright-field visible microscope image of a TPO initiated composite composed of a 60/40 wt% (Bis-GMA/TEGDMA) resin matrix containing 50 wt% of monodisperse 8 µm silica particles. The scale bar is equivalent to 8 µm. **b** An image of reactive group conversion over the identical region shown in (**a**). Areas coloured blue and red correspond to regions of relatively low and high conversion, respectively. **c** A coloured image of the aromatic (1608 cm^−1^) absorption band wavenumber position over the same region, where lower wavenumbers correspond to larger residual strain stored within the polymer (red regions). White regions correspond to spectra that either failed quality tests or where fits were poor. **d** A single Mid-IR spectrum (collected over 850–4000 cm^−1^, with a 2 cm^−1^ resolution) from one pixel following Mie scatter corrections of the same TPO initiated composite (solid black line). A non-Mie scatter corrected spectrum (offset in the *y*-axis for clarity) displays characteristic background oscillations and derivative shapes which distort spectral features (broken black line). (inset) Aromatic (1608 cm^−1^) and aliphatic (1637 cm^−1^) absorption bands which were used to calculate the degree of reactive group conversion and relative residual strain for each pixel within an image.

Figure 1a shows a bright-field image of a 60/40 wt% (Bisphenol-A-glycidyl-dimethacrylate/Triethyleneglycol-dimethacrylate, henceforth referred to as Bis-GMA/TEGDMA) resin matrix initiated with Lucirin TPO (TPO), containing a 50 wt% filler loading of highly monodisperse 8 µm (diameter) particles (shown as circles in 2D). The degree of reactive group conversion over the same 2D region is shown in Fig. 1b. Colour coding is used to highlight areas of relatively low (blue) and high (red) conversion corresponding to the resin matrix immediately surrounding and between filler particles. Spatial variation of residual strain within the vicinity of a filler particle (Fig. 1c) was determined semi-quantitatively by deconvolving the principal aromatic absorption band to obtain the peak position (~1608 cm^−1^) (Fig. 1d and inset) for the mid-IR spectrum in each pixel to produce images of wavenumber for every composite (Fig. 1c). Lower and higher wavenumbers are coloured red and blue, respectively, to illustrate higher and lower states of residual strain. Figure 1d also displays the effect of Mie scatter and subsequent corrections on a single exemplar spectrum. The dotted line displays a spectrum prior to correction. An oscillating background and a derivative feature located at ~1300 cm^−1^ are observed which may alter peak positions and shapes. The corrected spectrum (solid line) is free of the aforementioned scattering effects.

### Micro-scale spatial variation in reactive group conversion

**Fig. 2:**
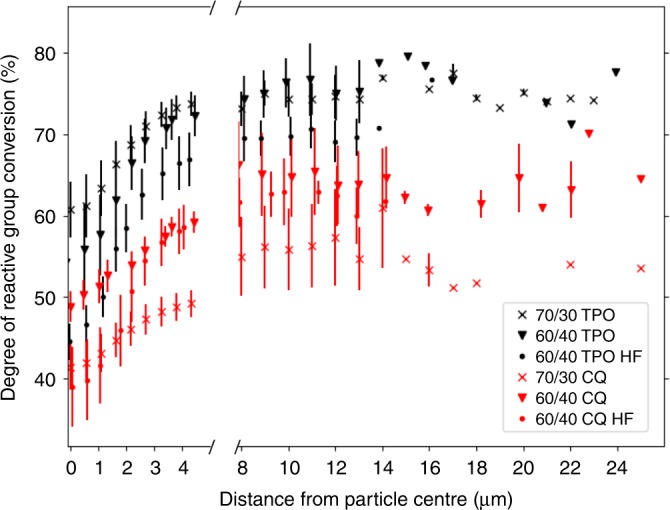
Inter-particulate variation in reactive group conversion. Degree of reactive group conversion as a function of distance from a filler particle centre. Radially averaged conversion (over all particles, centre to edge) is shown to the left of the axis break, while conversion at the midpoint between particle centres is shown to the right side. For both high filler (HF) fraction conditions, DC was quantified at shorter distances compared with other conditions as the particle packing density was higher. Error bars are reported as the standard deviation of radial and inter-particulate measurements. Jitter has been added to for clarity.

Figure 1b illustrates how the degree of conversion within the resin matrix varies as a function of distance from a filler particle centre. Radially averaged profiles of conversion over the area corresponding to the 2D particle projection for each specific composite are displayed to the left of the axis break in Fig. 2. It can be seen for all composites that the degree of conversion is lowest at the particle centre (projected in 2D) and increases, towards the particle edge approaching the interstitial resin matrix (*p* < 0.001). Unfilled neat resins do not display this pattern and demonstrate a more homogenous spatial distribution of reactive group conversion (Supplementary Fig. 1). Composites polymerised with the more efficient photo-initiator Lucirin TPO (TPO)^28^, demonstrate significantly greater conversion for identical resin matrix compositions compared with camphorquinone (CQ) based systems which polymerise at slower rates of conversion under identical irradiance (*p* < 0.001) (Supplementary Fig. 2). For 70/30 and 60/40 wt% blends, conversion is ~20 and 10% greater, respectively, when the system is polymerised with TPO for both filler loading fractions. The increase in conversion from the centre to the edge of a filler particle is ~15% and ~10% for TPO and CQ initiated systems, respectively, for all 50/50 wt% filler loadings, regardless of initial resin viscosity. Composites with a higher filler (HF) fraction demonstrate lower conversion at the particle centre (*p* < 0.001), but display similar values of conversion at the particle edge, compared with lower filled composites with an equivalent resin matrix composition.

Inter-particulate conversion for all composites, where conversion was measured at the midpoint between the centre points of two neighbouring particles for a range of separation distances, is shown to the right of the axis break in Fig. 2. A larger separation distance between local filler particles corresponds to increased conversion in the resin matrix until reaching a plateau. Offsets in inter-particulate conversion between identical blends but polymerised with different photo-initiator species are consistent with their corresponding radial profiles, with TPO initiated resins showing ~20 and 10% greater DC between filler particles for 70/30 and 60/40 wt% blends, respectively.

**Fig. 3:**
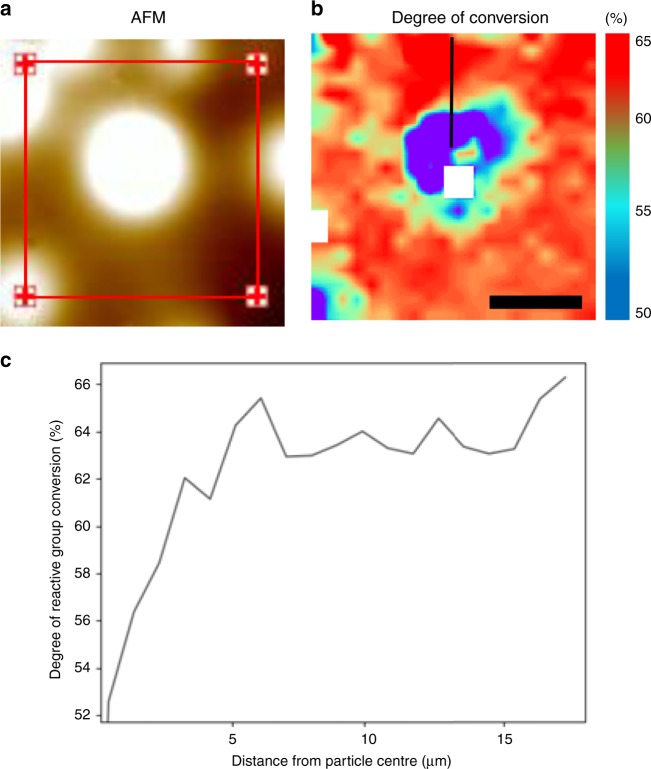
AFM-IR characterisation of inter-particulate reactive group conversion. **a** A region of a sample containing silica spheres embedded within a 60/40 wt% (Bis-GMA/TEGDMA) resin matrix initiated with CQ that was imaged using AFM-IR. **b** Spatial distribution of reactive group conversion over the same region of interest shown in (**a**). Red and blue regions correspond to higher and lower reactive group conversion while white pixels represent spectra which did not pass a quality test. The scale bar is equivalent to 8 µm. **c** Line transect, shown in (**b**), of reactive group conversion through the central silica sphere. Deconvolution of representative spectra obtained using AFM-IR are shown in Supplementary Fig. 3.

In addition to wide-field hyperspectral imaging combined with Mie scatter corrections, atomic force microscope infrared (AFM-IR) spectroscopy was used to confirm the inter-particulate pattern of reactive group conversion observed with wide-field mid-FTIR imaging. AFM-IR is an indirect approach exploiting photo-thermal effects. Figure 3a shows a region of a sample containing silica spheres embedded within a 60/40 wt% (Bis-GMA/TEGDMA) resin matrix initiated with CQ that was imaged using AFM-IR. Reactive group conversion, over the same area is illustrated in Fig. 3b. Lower conversion is observed over the region occupied by the silica sphere which increases into the neat resin matrix. A line transect from the centre of the silica particle into the resin matrix (Fig. 3c) shows a similar profile to other CQ initiated systems seen in Fig. 2.

### Residual strain

**Fig. 4:**
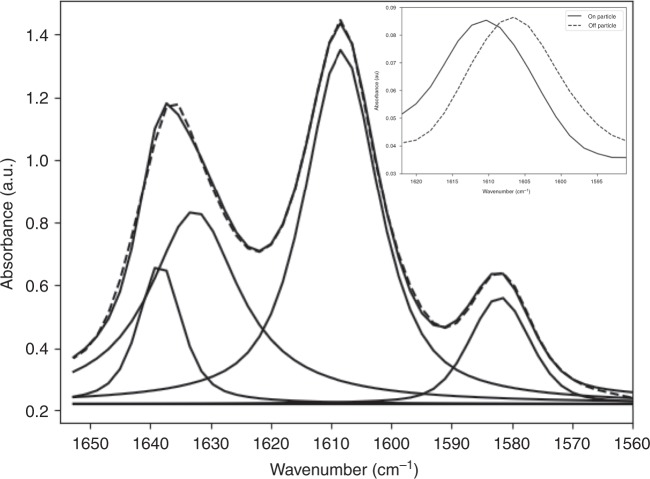
Residual aromatic strain analysis. Deconvolution of the aromatic (1581 and 1608 cm^−1^) and aliphatic (*cis* = 1632.5 cm^−1^, *trans* = 1638.5 cm^−1^) peaks to indirectly quantify residual strain. (Inset) The principal aromatic peak (1608 cm^−1^) shifts from a higher to a lower wavenumber value corresponding to ‘on’ and ‘off’ particle positions, respectively.

**Fig. 5:**
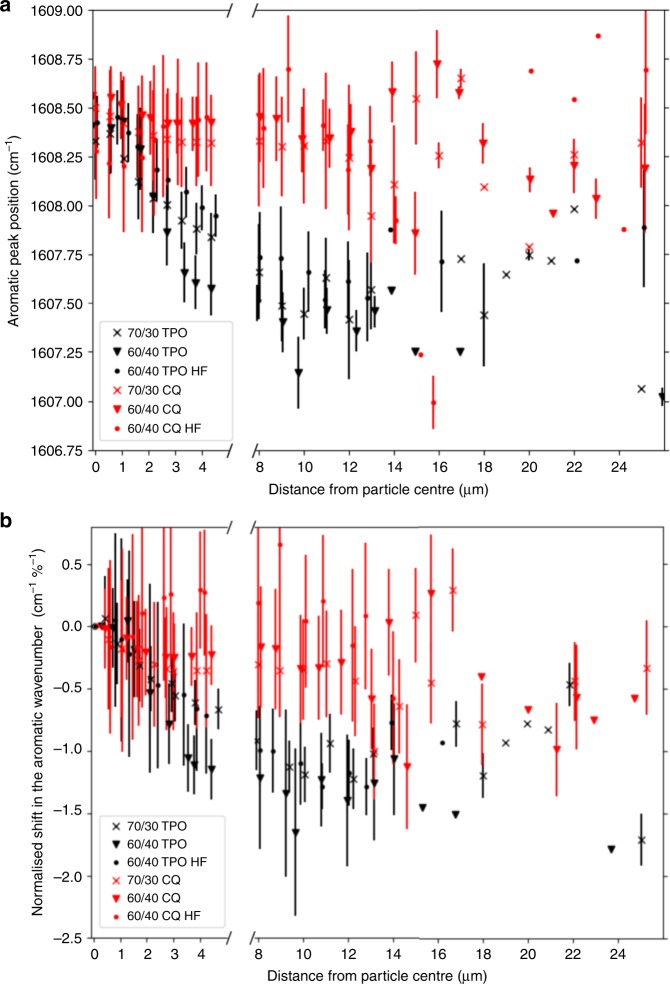
Inter-particulate residual aromatic strain. **a** The peak position for the absorption band corresponding to the aromatic group at the centre of the Bis-GMA monomer as a function of distance from a filler particle centre. Radially averaged wavenumber values (over all particles, centre to edge) are shown to the left of the axis break, while wavenumber values at the midpoint between particle centres are shown to the right. **b** The shift in the aromatic wavenumber (an increase in residual strain), normalised to the degree of reactive group conversion at each distance increment. Error bars are reported as the standard deviation of radial and inter-particulate measurements. Jitter has been added to for clarity.

Figure 1c displays the spatial variability of the deconvolved wavenumber peak value of the principal aromatic absorption band (~1608 cm^−1^, Fig. 4). Spectral deconvolution^29^ was used to identify shifts to lower wavenumber values in the principal aromatic absorption band (Fig. 4, inset) originating from the benzene groups at the centre of the Bis-GMA monomer. Strain in chemical bonds has been shown to correspond to a decrease in the observed wavenumber for specific absorption bands^30^. Greater aromatic wavenumbers (lower residual strain) are observed at the 2D particle centres (Fig. 1c) and decrease into the inter-particulate resin matrix. In Fig. 5a, radial averages of the wavenumber peak value of the aromatic absorption band are displayed to the left of the axis break while inter-particulate values are shown to the right. Radial averages for all composite samples show that at the 2D particle centre the principal aromatic band position is close to ~1608.4 cm^−1^ with small variations between blends. This value is very similar to the wavenumber of the aromatic absorption band in the Bis-GMA (liquid) monomer (Supplementary Fig. 4). The aromatic frequency shifts to lower wavenumbers towards the edge of the filler particle. We observed negligible wavenumber shifts into the inter-particulate resin matrix for CQ initiated systems (*p* = 0.729), while TPO based systems demonstrated statistically significant shifts to lower wavenumbers of ~0.8 and 1.7 cm^−1^ for 70/30 and 60/40 wt% formulations, respectively, (*p* < 0.001). For all composite formulations (with the exception of the CQ initiated 60/40 wt% high filler fraction composite which shows a slight increase in wavenumber), the decrease in wavenumber (an increase in bond strain) correlates strongly with increasing degree of conversion over the 2D projection of a given particle, with correlation coefficients of *r* ≤ −0.93 (*p* < 0.001).

Direct comparison of aromatic frequency shifts (residual strain) between composites is complicated by differences in reactive group conversion for each polymerising system. Normalising the aromatic peak shift, calculated from fitted spectral data, to the degree of conversion for each data point at a given separation distance (Fig. 5b) shows a similar relationship to the radial averages in Fig. 5a. In addition, TPO based systems that contained a greater proportion of the lower initial viscosity monomer TEGDMA (Supplementary Fig. 5), display a greater aromatic shift per converted reactive group. To the right of the axis break in Fig. 5a, it can be seen that differences in wavenumber shift with respect to photo-initiator chemistry and initial resin matrix viscosity are maintained at inter-particulate distances. The aromatic wavenumber for 60/40 wt% TPO composites decreases to 1606.9 ± 0.017 cm^−1^ at larger particle separation distances and correlates with increasing conversion (*r* ≈ −0.80, *p* < 0.001) up to separation distances of ~12 µm before conversion and strain plateau.

Some caution must be taken when interpreting subtle shifts in the frequency of absorption bands, particularly when spectra have been obtained with an FPA and spatial oversampling performed. To verify the observed 2D pattern of aromatic strain, additional synchrotron Fourier transform micro-spectroscopy measurements were undertaken employing point by point spectral acquisition using a pin-hole optical geometry at discrete locations within composites to eliminate possible spatial oversampling effects. The additional spectral data confirms that a wavenumber shift in the aromatic absorption band is still seen in the TPO initiated systems only (*p* < 0.001) (Supplementary Fig. 6 and Table 1).

**Table 1:** Summary of sample composition and corresponding sample codes.

Bis-GMA (wt%)	TEGDMA (wt%)	Photo-initiator	Resin/filler (wt%)	Sample code
70	30	CQ	50/50	70/30 CQ
70	30	TPO	50/50	70/30 TPO
60	40	CQ	50/50	60/40 CQ
60	40	TPO	50/50	60/40 TPO
60	40	CQ	40/60	60/40 CQ HF
60	40	TPO	40/60	60/40 TPO HF

Weight percentage (wt%) mixing ratios of Bis-GMA and TEGDMA monomers used to formulate each resin blend. Each mixing ratio was combined with a photo-initiator as either 0.2 wt% camphorquinone (CQ) with 0.8 wt% of its tertiary amine N,N-dimethylaminoethyl-methacrylate (DMAEMA) or 1 wt% Lucirin-TPO (TPO) photo-initiator. Silica filler particles were added in higher (HF) (40/60) and lower (50/50) wt% loadings to form composites.

## Discussion

In this study, we demonstrate how the presence of silica filler particles in a dimethacrylate polymer matrix induces spatial heterogeneity with respect to reactive group conversion and residual strains. Previous FTIR spectroscopy based studies by several groups have quantified DC in similar composite systems^18,22,31,32^, but to date have only been able to provide bulk measurements where the spatial resolution of the FTIR probe is significantly greater than inter-particulate distances, effectively averaging over values of conversion arising from resin within the immediate vicinity of filler particles and the inter-particulate resin matrix. Here, employing the brightness of a multi-beam synchrotron source coupled with a diffraction-limited IR wide-field imaging instrument and spatial oversampling enabled us to discern subtle differences in the micro-scale structure of composites at inter-particulate length scales.

Reactive group conversion and residual strain display an increase from the 2D centre to the particle edge (Figs. 2 and 5a) for all composite formulations. The observed pattern of conversion is not attributed to differential conversion as a function of sample thickness, given that the polished samples measured were ~10 µm thick, the effects of linear attenuation on conversion are considered to be negligible^23,33^. It has also been shown that gradients in conversion through the sample thickness manifest at depths exceeding 1 mm^25^. The most likely explanation for this relationship is the presence of a lower converted boundary layer^34–37^ storing less or no residual aromatic bond strain, encapsulating each filler particle, relative to the inter-particulate resin matrix. As each filler particle is viewed in 2D, the measured values of conversion and strain in a pixel represent the combined contribution of the values arising from the boundary layer and the inter-particulate resin matrix. At the 2D centre, the relative contribution of the boundary layer to the spectral signal is greatest, but towards the particle edge and away from the particle the inter-particulate resin matrix makes an increasing contribution to the sample composition and spectral signal and increases in DC and strain are observed. The formation of this boundary layer is attributed to reduced monomer mobility around the silica particle^18,36,38^, due to drag along the particle surface likely influenced by hydrogen bonding interactions (between the monomers and silica). This is supported by the homogenous spatial distribution of reactive group conversion observed in unfilled resins at these length scales (Supplementary Fig. 1). Lower monomer mobility will restrict the diffusion of propagating radical species during polymerisation, reducing conversion in these regions relative to the interstitial matrix. The inter-particulate pattern of conversion obtained using wide-field mid-FTIR imaging is not believed to be a result of Mie scattering effects given the application of state-of-the-art theoretical data corrections in addition to supporting AFM-IR measurements (Fig. 3), insensitive to Mie scattering, which confirm our observations. In addition, spectral deconvolution demonstrates that relative intensity changes in overlapping neighbouring peaks, i.e. the aliphatic absorption bands, do not artificially induce wavenumber shifts in the principal aromatic absorption band.

Away from the vicinity of a filler particle, conversion shows a small increase (~2–5%) for all composites up to a separation distance of ~12 µm before plateauing. The increase in conversion off of a particle at smaller separation distances (<12 µm) may be explained by considering differences in particle packing density and system mobility within the resin matrix during polymerisation. Smaller separation distances will correspond to greater packing densities and therefore represent regions where the impinging light has been attenuated to a greater extent compared with regions of lower packing density, reducing reactive group conversion. In addition, as the packing density decreases at greater separation distances, the resin phase will have greater mobility as a reduced proportion of the resin will exist as a boundary layer conferring increased conversion.

To date, the physico-mechanical and optical properties of composites have been shown to vary as a function of conversion and are often reported as a binary system, combining the properties of the resin bulk and that of the filler. However, a boundary layer with lower conversion will produce an additional region (including the filler phase and inter-particulate resin matrix) which will mismatch with the inter-particulate matrix with respect to elastic modulus, glass transition temperature, tensile and compressive strength, refractive index, thermal expansion, polymerisation shrinkage and polymerisation induced stresses^13,24,39–44^. The interaction between the resin matrix and the filler particles is likely to be further modified by silane surface treatment within commercial materials which will impact on monomer mobility and reactive group conversion near to the particle surface.

The generation of internal stresses in these materials are often considered in terms of viscous flow of the bulk during polymerisation, varying as a function of terminal conversion and polymerisation rate^45^. It is accepted that for dimethacrylate based polymers, immediate shrinkage strain is the result of Van der Waals inter-molecular spacing being exchanged for that of carbon–carbon single bonds as the polymer forms^46^. Although the aromatic core of Bis-GMA is the stiffest component of the molecule, it is discrete with respect to conversion and cross-linking reactions. Other bonds in the monomer will be subject to strain before strains in the aromatic groups are detectable^47,48^, but as the IR band corresponding to this structure is very well defined, subtle differences in the peak position can be elucidated.

Composites initiated with the more efficient photo-initiator TPO^28^ display greater conversion and strain (a decrease in wavenumber) over a filler particle compared with CQ systems which display reduced conversion and negligible strain (*p* < 0.001). In addition, within each photo-initiator system, composites with a resin matrix containing a greater fraction of the lower viscosity TEGDMA monomer (see Supplementary Fig. 5) also display greater strain (Fig. 5a). This is consistent with the literature, which has shown that TPO is a more efficient photo-initiator than CQ^28^ and achieves greater terminal conversion and maximum conversion rate^47^ (Supplementary Fig. 2), up to an order of magnitude for the latter^48^. Similarly, a less viscous resin matrix confers greater mobility for propagating radical species during polymerisation^46,49^. Making correlations, however, between residual strain and polymerisation variables known to influence terminal conversion and the rate of conversion are complicated by differences in the degree of conversion for each composite. A more converted polymer will generate greater internal stresses compared with a lower converted material that has been polymerised under similar conditions^48^. Normalising the decrease in the aromatic wavenumber with respect to DC_*i*_ (where DC_*i*_ represents the measured value of conversion at the *i*th increment of particle separation) and plotting against distance demonstrates that the generation of aromatic strain (residual strain within the polymer structure) is ranked by photo-initiator chemistry with sub-sets of resin matrix viscosity (Fig. 5b). We have previously reported that Bis-GMA/TEGDMA systems initiated with TPO and a higher content of TEGDMA typically achieve greater conversion rates during polymerisation conferring rapid chain extension (measured using X-ray scattering) within polymer segments which may be stored as residual strain^48^. Under similar photo-polymerisation conditions, CQ based systems displayed negligible or no residual strain^48^. Correlating the aromatic strain detected in this study, with the polymer chain segment extension for identical resin systems under very similar photo-polymerisation conditions^48^ demonstrated a strong linear relationship (Supplementary Fig. 7). It is therefore suggested that rapid chain extension facilitated by efficient radical generation and relatively fast reactive group conversion, due to TPO initiation, is the mechanism which results in the aromatic strain reported in this study.

Composites with a 60/40 wt% loading of filler display similar radial and inter-particulate profiles, with respect to conversion and aromatic strain, in comparison with lower filled counterparts. As the filler particles are uniform, individual particles will produce similar patterns in conversion as a function of packing density, which will influence light attenuation and relative monomer mobility. However, as larger particle separation distances do not exist in higher filler load conditions, the composite cannot achieve greater conversion. Therefore, the average value of conversion for a high-filled composite is relatively lower, which is consistent with reported bulk measurements.

In this study, composites were prepared as thicker samples and polished as this was a feasible way to fabricate samples thin enough to conduct Mid-IR transmission measurements while maintaining the packing density of filler particles. Although out of plane particles were present during polymerisation and have subsequently been removed, Figs. 1b, c and 2 demonstrate that the variability in conversion and strain is predominantly limited to the radius of the filler particle and plateaus moving almost immediately off-particle. Neighbouring particles will therefore have little effect on the radial profiles (as can be seen in neighbouring 2D particles within the image plane of Figs. 1b and c) and any minor variability is already accounted for in the accompanying error bars. The magnitudes of the reported error bars are relatively small and do not affect the interpretation of the observed trends in conversion and aromatic bond strain.

The micro-structure of light activated resin composites has been studied with micron-scale spatial resolution using synchrotron-based FTIR wide-field imaging at inter-particulate length scales. The presence of filler particles in a resin matrix has been shown to introduce inter-particulate spatial heterogeneity with regard to both reactive group conversion and residual bond strain, which has been hypothesised^36,37^ but not demonstrated in any polymer-composite system to date. Heterogeneity close to a particle is believed to originate from a lower converted and reduced residual strain boundary layer, which encapsulates each particle. At inter-particulate length scales, increases in conversion and strain are attributed to reduced attenuation of impinging light and greater monomer and radical mobility within the resin matrix. Greater conversion, achieved through photo-initiator chemistry, resin matrix composition or increased filler particle separation, correlates to greater total aromatic bond strain in the cross-linking monomer. However, the generation of residual bond strain appears to be more sensitive to the relative differences in conversion rate.

Our findings are applicable to a wide range of polymer-composite materials and demonstrate how resin matrix composition, filler particles and relative differences in conversion rate may impact on the final micro-structure of a composite material. This information bridges a fundamental gap in the understanding of composite systems, providing valuable links between material composition, filler-matrix interfaces and reported bulk properties.

## Methods

### Preparation of experimental resin composites

Experimental composites were prepared by combining monodispersed filler particles with dimethacrylate monomers proportioned to provide differences in matrix viscosity and containing different photo-initiator chemistries to modify photo-polymerisation kinetics. The dimethacrylate monomers, bisphenol-A-glycidyl-dimethacrylate (Bis-GMA) and triethyleneglycol-dimethacrylate (TEGDMA) (Sigma-Aldrich, Dorset, UK) were proportioned in 70/30 and 60/40 (Bis-GMA/TEGDMA) weight percentage (wt%) ratios and combined to produce 10 g mixes. The viscosity of the blend decreases with the proportion of TEGDMA (Supplementary Fig. 5). The monomer blends were combined with a photo-initiator as either 0.2 wt% camphorquinone (CQ) with 0.8 wt% of its tertiary amine N,N-dimethylaminoethyl-methacrylate (DMAEMA) or 1 wt% Lucirin TPO (Sigma-Aldrich, Dorset, UK) to introduce extremes in the rate of reactive group conversion. The proportioned monomers and photo-initiators were homogenised in a glass beaker using a magnetic stirrer at 50 ± 1 °C for 30 min in dark conditions. Non-silanized, 8 µm (diameter, coefficient of variation <10%) silica micro-spheres (Cospheric, Santa Barbara, CA, USA, refractive index = 1.54) were added to the resin blends in either 50/50 or 40/60 wt% ratios and mixed to form composites, using a high-speed mixing machine (SpeedMixer™ DAC 150.1 FVZ-K, Synergy Devices Limited, Buckinghamshire, UK) for 5 min at 1000 rpm. The compositions for the filled resin blends are summarised in Table 1.

For each composition, 0.01 mL of (uncured) composite was applied to the surface of a calcium fluoride (CaF_2_) disc (30 mm × 1 mm (diameter × thickness), UV grade polished window, Crystan, Dorset, UK), and pressed with a microscope cover slide to an approximate thickness of 100 µm. Composites were photo-polymerised using an EMS Swiss master light curing unit (EMS OPTIDENT, Electro Medical Systems, Nyon, Switzerland) which was placed normal to and in contact with the cover slide, illuminating the composite for 60 s at an intensity of 300 mWcm^−2^ over a spectral range of 390–550 nm. SiC paper (P4000, Agar Scientific, Stansted, Essex, UK) was used to polish polymerised composites to a thickness of ~10 µm, leaving a monolayer of silica particles, to allow for the transmission of mid-infrared light. The resultant resins were stored in dark conditions in sealed containers at 4 ± 1 °C prior to further use.

### Synchrotron-based Fourier transform mid-infrared wide-field imaging

**Fig. 6:**
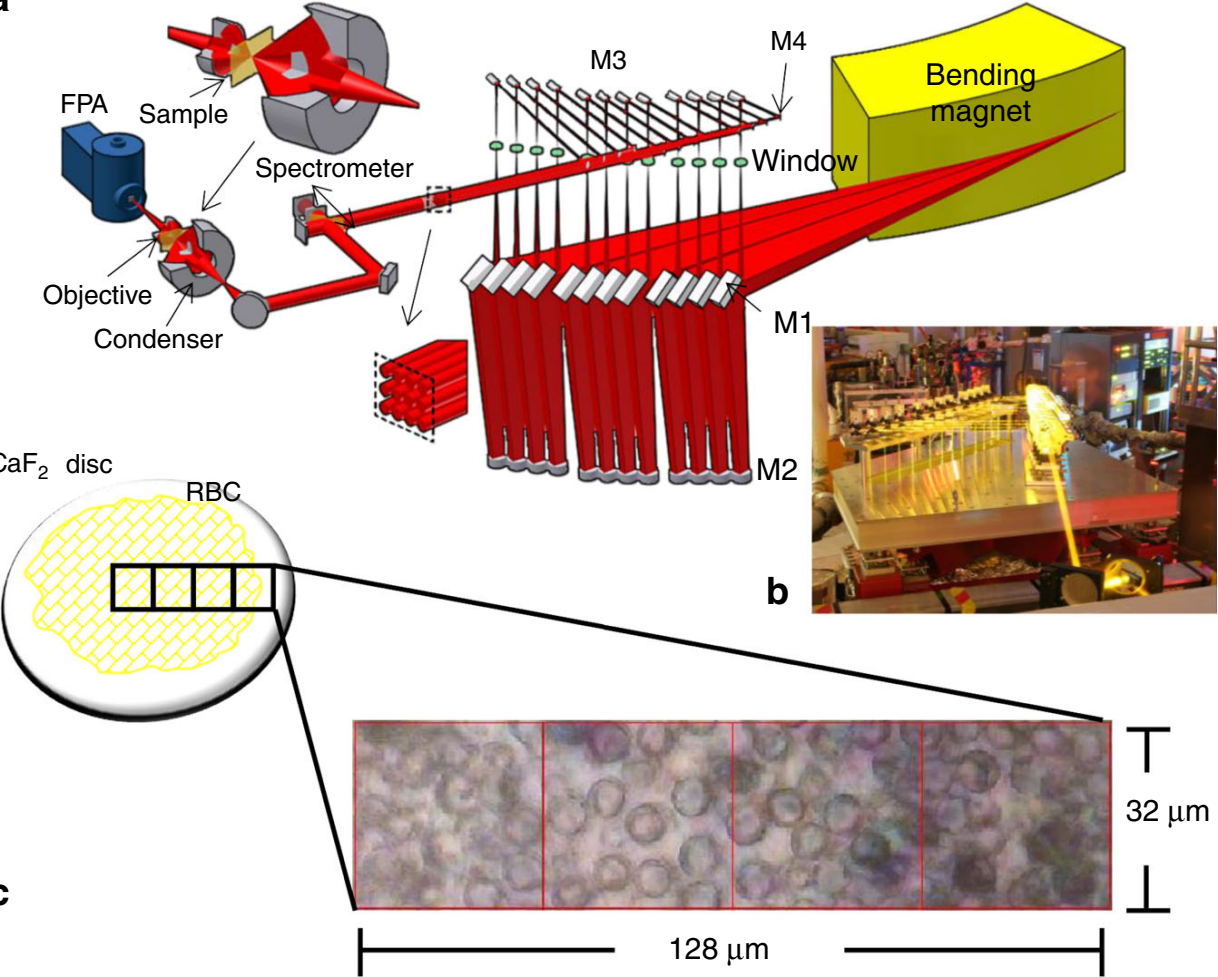
Experimental schematic of mid-IR wide-field imaging of polymer composites. **a** Schematic of the IRENI beamline (Synchrotron Radiation Centre, Wisconsin, USA), where M1–M4 are mirror sets (adapted from Nasse et al.^27^, with permission). **b** Long-exposure photograph showing the combination of 12 individual beams to form a 3 × 4 matrix illuminating a focal plane array (FPA). **c** A bright-field visible microscope image of a 60/40 wt% TPO initiated resin based composite (RBC) containing 8 µm monodisperse spherical silica filler particles.

High-resolution synchrotron Fourier transform mid-infrared (sFTIR) images were taken of each composite formulation at the IRENI (InfraRed Environmental Imaging) beamline (Fig. 6) (Synchrotron Radiation Centre (SRC), University of Wisconsin-Madison, Wisconsin, USA)^27^. Radiation from the synchrotron storage ring was extracted via a bending magnet into a 320 mrad (horizontal) by 25 mrad (vertical) swath, resembling a fan of radiation. This swath was subsequently decomposed into 12 independent beams of synchrotron light, which were collimated and combined side by side into a 3 × 4 matrix using an array of toroidal, plane and parabolic mirrors. The beam matrix illuminates a 60 × 40 µm^2^ region on an MCT (Mercury Cadmium Telluride) focal plane array (FPA, Santa Barbara) detector, housed within a Hyperion 3000 IR microscope (Bruker Optics) coupled with a Bruker 70 IR spectrometer. Transmission measurements were performed using a ×74 magnification objective (Ealing, Thermo Scientific) with a numerical aperture (NA) of 0.65 and a ×15 condenser objective (NA = 0.5). This optical configuration coupled with spatial oversampling allowed for diffraction-limited imaging (spatially) over the entire mid-IR spectral range (2.5–10 μm), and provides an effective pixel size of 0.54 µm × 0.54 µm at the sample plane. Hyperspectral data were collected with 2 cm^−1^ spectral resolution and 128 scans were co-added for signal averaging.

The microscope was equipped with an automated X–Y stage, enabling mosaic IR imaging. A liquid nitrogen-cooled FPA detector was set to 64 × 64 pixels; therefore, images of the composite were collected in tile mosaics, where each single tile was equivalent to an illuminated area of 32 × 32 μm^2^ (64 × 64 pixels). Larger images were constructed from the combination of several tiles, up to a maximum of eight, recorded sequentially. A single tile includes 4096 individual FTIR spectra (each pixel contains a mid-IR spectrum and all spectral bands were collected simultaneously), with a measurement time of ~5 min per tile. Images were collected from the centre of each composite/CaF_2_ disc using the commercial Bruker OPUS software package (version 6.5, Bruker Optics) in images consisting of 4, 6 or 8 tiles. For each FTIR image, a bright-field image was collected over the same area in order to locate the centre of each filler particle for analysis. Measurements were carried out in a nitrogen filled atmosphere at 23 ± 1 °C within a sealed Plexiglas container.

### Mie scatter corrections

The ME-EMSC algorithm is an iterative algorithm that retrieves pure absorbance infrared spectra from highly scatter-distorted measured spectra^50–53^. In each iteration step, the pure absorbance spectrum is updated and then used for calculating the complex refractive index. The complex refractive index serves as an input for calculating the scatter-distorted spectrum. Modelling of the scatter-distorted spectrum is done by a subspace model employing the van de Hulst approximation to the rigorous Mie theory for a parameter space for the refractive index and size of the spheres^54^. Prior to ME-EMSC correction, raw spectra were subjected to a basic EMSC correction to filter out background spectra or data with no Mie scattering features. Filtering the input spectra was done by applying a basic EMSC (up to the linear term) and removing spectra with a low optical path length and with low residuals in the basic EMSC model. A low optical path length indicates a weak absorbance signal, i.e. that the spectrum is from the background and not the sample. Further, when there is no Mie scattering signals present in the measured absorbance spectra, the residuals from a basic EMSC are expected to be relatively low, since the basic model describes all measured features well. These spectra do not need a scatter correction by the ME-EMSC and are sufficiently corrected by a basic EMSC. Between 11 and 100% of the raw hyperspectral data required Mie correction, while the remaining had either too low signal strength, or no Mie scattering features present in the spectra.

Spectra still demonstrating strong Mie scattering effects were passed to the ME-EMSC algorithm. The ME-EMSC requires as input parameters the radius of the Mie scatterer *a* and the real and constant part of the refractive index *n*_0_, which is needed in the subspace model. Refractive index values for each resin matrix composition were obtained for monomer and polymer states using a refractometer (J257, RUDOLPH RESEARCH ANALYTICAL, NJ, USA). For most of the raw spectra, the Mie correction was performed with initialisation parameter *a* in the range 6–10 µm, and *n*_0_ in the range 1.1–1.6. For some of the spectra, the range for *a* was set to 4–8 µm with *n*_0_ 1.1–1.4. The number of principal components included in the model varied from 10 to 14. To initialise the algorithm, reference spectra with low scatter signals were used. Reference spectra were estimated by taking the average of either all or a selection of the raw spectra, and performing a rubber band correction. As the reference spectrum is only used in the initialization of the algorithm, the rubber band correction is adequate for correcting the mean spectrum. For some spectra, a down-weighting of the inactive region was applied. A basic EMSC was also employed subsequent to the ME-EMSC model as a quality control of the corrected spectra. If the Mie scatter features are not completely removed due to inadequate initialization parameters, the residuals from a basic EMSC are relatively high. Spectra with high residuals from this basic EMSC were sent back to the ME-EMSC with adjusted parameters. Of the raw spectra which were passed on to the ME-EMSC algorithm, between 86 and 100% were successfully corrected.

### Atomic force microscopy infrared (AFM-IR) spectroscopy

Atomic force microscopy infrared (AFM-IR) spectroscopy, which obtains IR spectral data indirectly and is therefore not susceptible to the spectral distortions associated with light scattering^55,56^ by spherical particles in transmission mid-IR spectroscopy, was used to confirm that the spatial distribution of inter-particulate conversion was not an effect of resonant Mie scatter. Nanoscale Mid-Infrared spectroscopy maps were taken on a CQ based 60/40 wt% (Bis-GMA/TEGDMA) composite sample with a 50/50 wt% filler fraction irradiated at 300 mWcm^−2^ for 60 s, using a NanoIR2 system (Anasys Instruments, CA, USA). AFM-IR maps were collected in contact mode at a scan rate of 0.04 Hz using a gold-coated silicon nitride probe (Anasys Instruments, 0.07–0.4 N m^−1^ spring constant, 13 ± 4 kHz resonant frequency). An optical parametric oscillator was used as the source of IR radiation incident on the sample, which was subjected to 10 ns pulses at a repetition rate of 1 kHz. The amplitude of infrared induced cantilever oscillations were mapped using 32 co-averages per 1024 points per 300 scan lines over a region of 20 × 20 µm^2^. Mid-IR spectra were collected between wavenumbers of 1550 and 1800 cm^−1^ with a 4 cm^−1^ resolution using 1024 co-averaged spectra per point. Laboratory based FT-MIR was used to obtain spectra of the composite prior to photo-polymerisation, i.e. in the monomer form. Bulk measurements of the uncured composite were collected using a Nicolet 6700 spectrometer (Thermo Scientific, Warrington, UK). Spectra were collected in ATR mode using a white light source and an InGaAs detector over a spectral range of 800–4000 cm^−1^ with a 4 cm^−1^ spectral resolution. Line transects were taken through regions of interest corresponding to the location of filler particles to confirm the spatial distribution of conversion obtained using synchrotron-based Fourier transform mid-infrared wide-field imaging.

### Degree of reactive group conversion calculation

The degree of reactive group conversion (DC) for each single pixel spectrum in a given image was calculated from the normalised percentage decrease in the area of the aliphatic absorption band (1637 cm^−1^), corresponding to the aliphatic C=C stretching frequency, relative to the monomer state. During polymerisation, aliphatic C=C bonds are converted to single bonds, i.e. C–C, producing a decrease in the intensity of the aliphatic band. Aliphatic absorption peaks were normalised to the aromatic C=C stretching band (1608 cm^−1^), used as an internal standard in these systems^29^, to correct for variations in sample thickness (Eq. (1)).

1 $${\mathrm{DC}} = \left( {1 - \frac{{{\mathrm{C}}\! =\! {\mathrm{C}}_{{\mathrm{aliphatic}}}^{\mathrm{p}}/{\mathrm{C}}\! =\! {\mathrm{C}}_{{\mathrm{aromatic}}}^{\mathrm{p}}}}{{{\mathrm{C}}\! =\! {\mathrm{C}}_{{\mathrm{aliphatic}}}^{\mathrm{m}}/{\mathrm{C}}\! =\! {\mathrm{C}}_{{\mathrm{aromatic}}}^{\mathrm{m}}}}} \right)100$$

Here C=C_aliphatic_ and C=C_aromatic_ refer to the area of the 1637 and 1608 cm^−1^ absorption bands, respectively, in the monomer (m) and polymer (p) forms. FTIR spectroscopy images were analysed using IRyidys (www.iryidys.com), an in-house programme generated at the SRC, which runs on the commercial software package IGOR PRO (version 6.3.5.5, WaveMetrics Inc.). Each image was integrated over the C=C aromatic (1608 cm^−1^) and aliphatic (1637 cm^−1^) absorption bands, respectively, within regions encompassed by the construction of linear baselines for each peak from points taken in the depressions adjacent to the peaks, and the degree of conversion was determined using Eq. (1) for each spectrum/pixel. Laboratory based FTIR was used to obtain baseline spectra of the composite prior to photo-polymerisation, i.e. zero reactive group conversion. Bulk measurements of the uncured composite were collected using a Nicolet 6700 spectrometer (Thermo Scientific, Warrington, UK). Spectra were collected in ATR mode using a white light source and an InGaAs detector over a spectral range of 800–4000 cm^−1^ with a 2 cm^−1^ spectral resolution. The ImageJ radial profile plugin was used to radially average DC, over a radius of ~4.5 µm at 0.54 µm increments, for all particles in an image. Conversion was also measured at inter-particulate distances, by obtaining conversion values at the midpoint between neighbouring particles (centre to centre distance) for all particles within an image and rebinning values within multiples of the pixel size to aid statistical analysis. Error bars for the radial and inter-particulate profiles were calculated as the standard deviation of conversion values at each distance from a particle centre.

### Measurements of residual strain

Residual strain was visualised indirectly from positional fluctuations of the principal aromatic (1608 cm^−1^) absorption band, corresponding to the aromatic C=C stretching frequency originating from the benzene rings at the centre of the Bis-GMA monomer. The aromatic group is ideally located to quantify bond strain and has a distinct chemical signature in the mid-IR region. Curve fitting was performed using the Lmfit package^57^ (version 1.0.0, Zenodo). Mie corrected spectra, in the 1550–1650 cm^−1^ region, were fit with Voigt models for the two aromatic rings (~1608 and 1588 cm^−1^). The vinyl stretch (1637 cm^−1^) was fit with two constituent peaks corresponding to the s-*cis* and s-*trans* rotational isomers^29^. The position of the s-*cis* and s-*trans* peaks were obtained from the curve fitting of Bis-GMA and TEGDMA monomers and fixed at 1632.5 and 1638.5 cm^−1^, respectively, to avoid coupling of the two peaks. The 1608 cm^−1^ aromatic band was chosen to indirectly visualise strain, instead of the 1588 cm^−1^ band, due to a greater signal-to-noise ratio. Images for each composite sample were constructed from the fitted aromatic peak positions (~1608 cm^−1^) from each spectrum and radial and inter-particulate profiles were calculated from particle centre locations. Error bars are reported as the standard deviation of all pixels/fitted values within a given distance bin from a particle centre.

### Statistical analysis

All experimental results are reported in figures and tables as the mean ± standard deviation or mean with propagated errors. A series of two-way, one-way ANOVA and post hoc Tukey tests (*α* = 0.05) were used to identify significant differences in conversion and the frequency of the principal aromatic absorption band for on and off filler particle locations between samples with identical resin matrix composition using either CQ or TPO photo-initiator systems. An unpaired, one sided with equal variance *t*-test was used to ascertain differences in reactive group conversion rate between CQ and TPO initiated systems (Supplementary Fig. 2). Linear regression was performed to ascertain the relationship between polymer chain segment extension (secondary analysis of data from ref. ^48^) and variation in the aromatic absorption frequency (Supplementary Fig. 7) as a function of irradiance protocol. All statistical analysis was performed using IBM® SPSS Statistics (version 25, IBM Corporation).

## Supplementary information

Supplementary Information

## Data Availability

Raw data were generated at the Synchrotron Radiation Center and the Canadian Light Source. Derived data supporting the findings of this study require significant computational processing to correct for Mie scatter effects and are therefore available from the corresponding author upon request.
