# Supplementary material for: Origin of micro-scale heterogeneity in polymerisation of photo-activated resin composites
Source: Nat Commun. 2020 Apr 15;11:1849. doi: 10.1038/s41467-020-15669-z (PMC7160210; doi:10.1038/s41467-020-15669-z)
Supplement: Supplementary file 1 — Supplementary Information [file 41467_2020_15669_MOESM1_ESM.pdf]

Supplementary Information

Origin of Micro-Scale Heterogeneity in Polymerisation of Photo-Activated Resin Composites

Slobodan Sirovica et al.

Supplementary Methods

FT-NIR Spectroscopy Measurements

Fourier transform near infra-red (FT-NIR) spectroscopy was used to quantify the total degree of and rate of reactive group conversion between 60/40 wt% (Bis-GMA/TEGDMA) blends initiated with either CQ or TPO. Optical (emitting and receiving) fibers (0.6 mm core diameter) (Hellma Analytics, Essex, UK) were placed either side of the liquid resin sample, which was held within a polyvinylsiloxane annulus (11 mm diameter and 1 mm in depth), at a 45° incline relative to the sample face and connected to a Nicolet 6700 spectrometer (Thermo Scientific, Warrington, UK). During photo-polymerisation, NIR spectra were collected in transmission mode using a white light source and an InGaAs detector. Real-time NIR spectra (4000−10000 cm^{-1}) were collected 10 s prior to irradiance and thereafter during polymerisation in transmission mode (4 cm^{-1} spectral resolution) with an integration time of 0.3 s, whilst photo-polymerising the resin sample at an irradiance of 300 mW cm^{-2}. Three measurements were conducted for each unique blend for both irradiance protocols. Data were baseline corrected using Omnic software (Omnic Specta software, version 8.0, Thermo Fisher Scientific, Oxford, UK). Reactive group conversion was determined from the percentage decrease in the aliphatic $C=CH_2$ IR absorption band (6164 cm^{-1}) i.e. the conversion of C=C to C-C bonds, located within the methylene functional end groups of the Bis-GMA and TEGDMA monomers. The rate of conversion was calculated as the first derivative of conversion with respect to time.

Mid-FTIR (ATR) Spectroscopy Measurements

Laboratory based mid-FTIR spectroscopy was used to obtain spectra of Bis-GMA monomer to confirm the position of the principal aromatic absorption band. Measurements were collected using a Nicolet 8700 spectrometer (Thermo Scientific, Alberta, Canada). Spectra were collected in ATR mode using an infrared light source and an InGaAs detector over a spectral range of 350 to 7400 cm^{-1} with a 1.92 cm^{-1} spectral resolution and 256 scans were co-added for signal averaging. Liquid monomer was applied directly to the diamond ATR crystal whilst undertaking measurements.

Viscosity Analysis

The viscosities of Bis-GMA/TEGDMA wt% monomer blends, as a function of Bis-GMA content (20, 30, 40, 50, 60, 70 and 100 wt%), were measured using a rheometer (AR 2000, TA Instruments, Ontario, Canada). Measurements for each unique monomer blend composition (n=20) were performed at 25°C± 0.1°C using a Peltier controlled temperature plate at a shear rate of 51.6 s^{-1}.

Pin-Hole Point Synchrotron FTIR Measurements (No Spatial Oversampling)

To confirm that the same pattern of observed shifts in the peak position of the aromatic absorption band exists without oversampling, further spectral data was acquired using a collection mode without any spatial oversampling. Diffraction limited synchrotron Fourier transform mid-infrared (SR-FTIR) spectroscopy point measurements were conducted for TPO and CQ based 60/40 wt% (Bis-GMA/TEGDMA) composites using the Mid-IR (01B1-1) beamline instrument (Canadian Light Source, Saskatoon, Canada). The synchrotron light source illuminates a single point liquid nitrogen cooled MCT (Mercury Cadmium Telluride) detector housed within a Hyperion 3000 IR microscope (Bruker Optics) coupled with a Bruker 70v/s IR spectrometer (Bruker optics Ltd., ON, Canada) over a spectral range of 560 – 6000 cm^{-1}. A pin-hole optical configuration allowed for diffraction limited spectral acquisition with a beam size of approximately 6 µm × 6 µm at the sample plane at 1608 cm^{-1}. Spectral data were collected at points located either on or off particle and discrete from neighbouring acquisition points to avoid spatial oversampling. Spectra were collected with 1 cm^{-1} spectral resolution, which is smaller than the observed shift in the aromatic wavenumber, and 128 scans were co-added for signal averaging.

Supplementary Fig.1: Visualisation of reactive group conversion in an unfilled resin

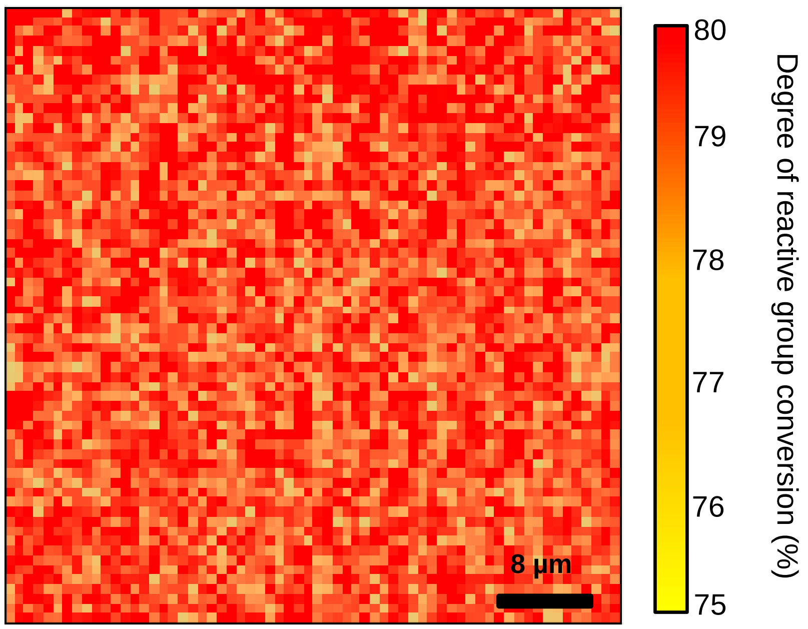

Supplementary Fig.1. An image of reactive group conversion for an unfilled 60/40 wt% (Bis-GMA/TEGDMA) resin matrix initiated with TPO.

Supplementary Fig.2: Resin polymerisation kinetics as a function of photo-initiator chemistry

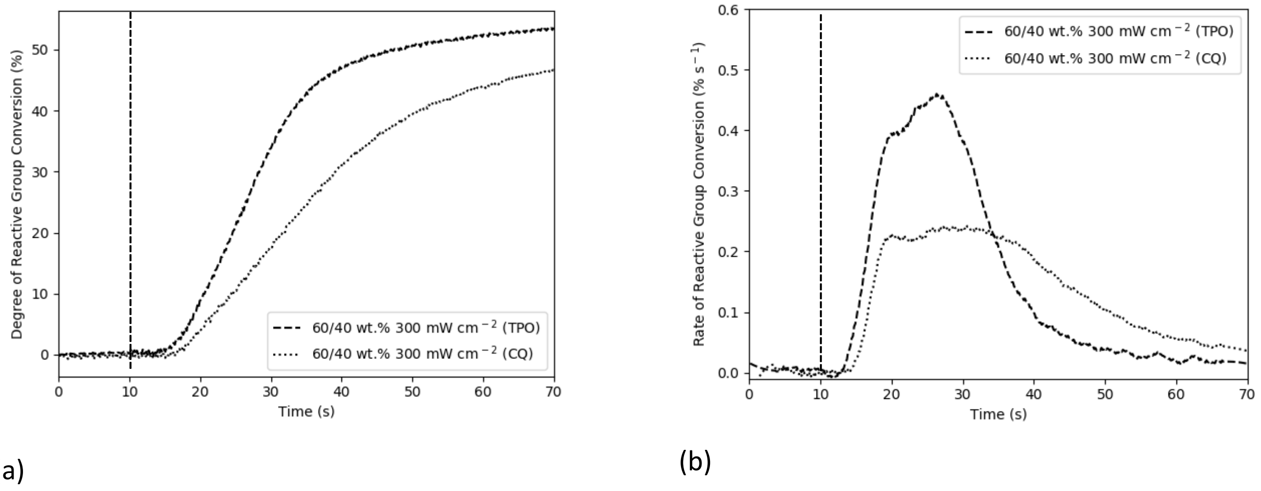

Supplementary Fig.2. (a) Degree of reactive group conversion, as a function of time, for 60/40 wt% (Bis-GMA/TEGDMA) blends initiated with either CQ or TPO throughout 60 s of irradiance at 300 mW cm^{-2} and the corresponding (b) rate of reactive group conversion. Vertical broken lines indicate the onset of irradiance.

Supplementary Fig.3: Representative AFM-IR spectra

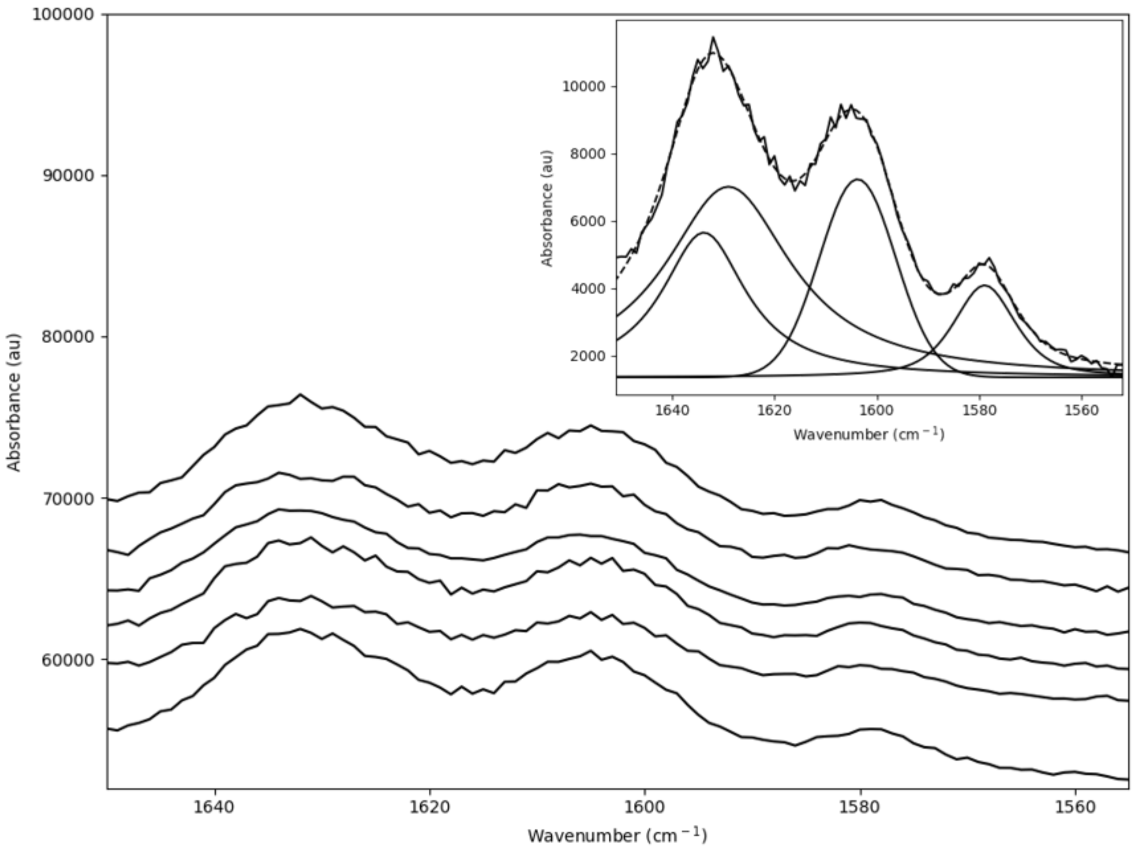

Supplementary Fig.3. Several mid-IR spectra, for a 60/40 wt% (Bis-GMA/TEGDMA) resin matrix containing 50 wt% of monodispearse 8 µm silica particles, obtained using AFM-IR spectroscopy. Spectra correspond to on and off particle locations. (inset) Deconvolution of the underlying aromatic (1581 and 1608 cm-1) and aliphatic (cis = 1632.5 cm^{-1}, trans = 1638.5 cm^{-1}) absorption bands.

Supplementary Fig.4: Representative mid-IR spectrum of Bis-GMA close to the aromatic absorption band frequency

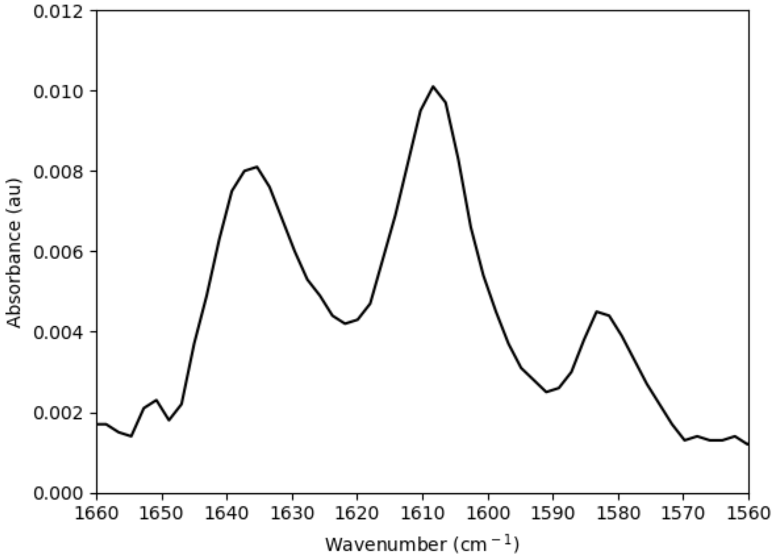

Supplementary Fig.4. Mid-IR spectrum of Bis-GMA, displayed between 1560-1660 cm^{-1}. The principal aromatic peak centre is located at ~1608.4 cm^{-1}.

Supplementary Fig.5: Resin viscosity as a function of comonomer ratio

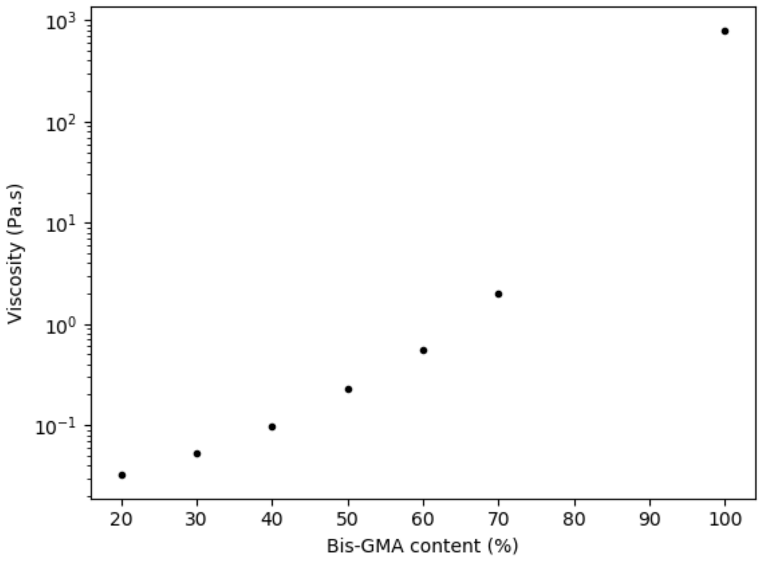

Supplementary Fig.5. Viscosity of Bis-GMA/TEGDMA wt% monomer blends as a function of the Bis-GMA content (wt%). Error bars, reported as the standard deviation of repeat measurements (n=20), are shown but are smaller than the size of the plotting symbols.

Pin-Hole Point Synchrotron FTIR Measurements (No Spatial Oversampling)

Figure 6a shows a visible image of a region containing three 8 µm (diameter) silica spheres within a 60/40 wt% (Bis-GMA/TEGDMA) resin matrix which has been initiated with the faster polymerising TPO photo-initiator. Overlaid are several on and off particle points from where spectral data was obtained, spatially discrete from the other sampling points. A similar plot is shown for a resin composite system initiated with CQ (Fig. 6b). Representative second derivative spectra corresponding to on and off particle locations demonstrate higher (~1608 cm^{-1}) and lower (~1606 cm^{-1}) wavenumber values respectively for the TPO based systems, whilst CQ based systems do not display a shift in the aromatic wavenumber. Mean values for the aromatic absorption band position using point measurements for on and off particle locations for the TPO and CQ based resin composites are given in Supplementary Table 1.

Correlating Aromatic Strain to Polymer Chain Segment Extension

Figure 7 illustrates the relationship between polymer chain extension (previously measured by the authors using X-ray scattering) and the observed wavenumber shift (aromatic strain) within identically composed 70/30 and 60/40 (Bis-GMA/TEGDMA) wt% systems initiated with either CQ or TPO as the photo-initiator and photo-polymerised under very similar irradiance conditions. The shift in the aromatic wavenumber is shown as a function of chain extension and it can be seen that greater chain extension, itself a function of conversion rate, confers larger shifts in the aromatic wavenumber i.e. greater aromatic strain. The highly linear relationship and strong correlation between chain extension and wavenumber shift (R^2 = 0.923) demonstrates that the origin of the observed wavenumber shift is likely due to the transfer of strain from extended polymer chain segments and is not an effect of spatial oversampling.

Supplementary Fig.6: Pin-hole point synchrotron FTIR measurements of aromatic strain

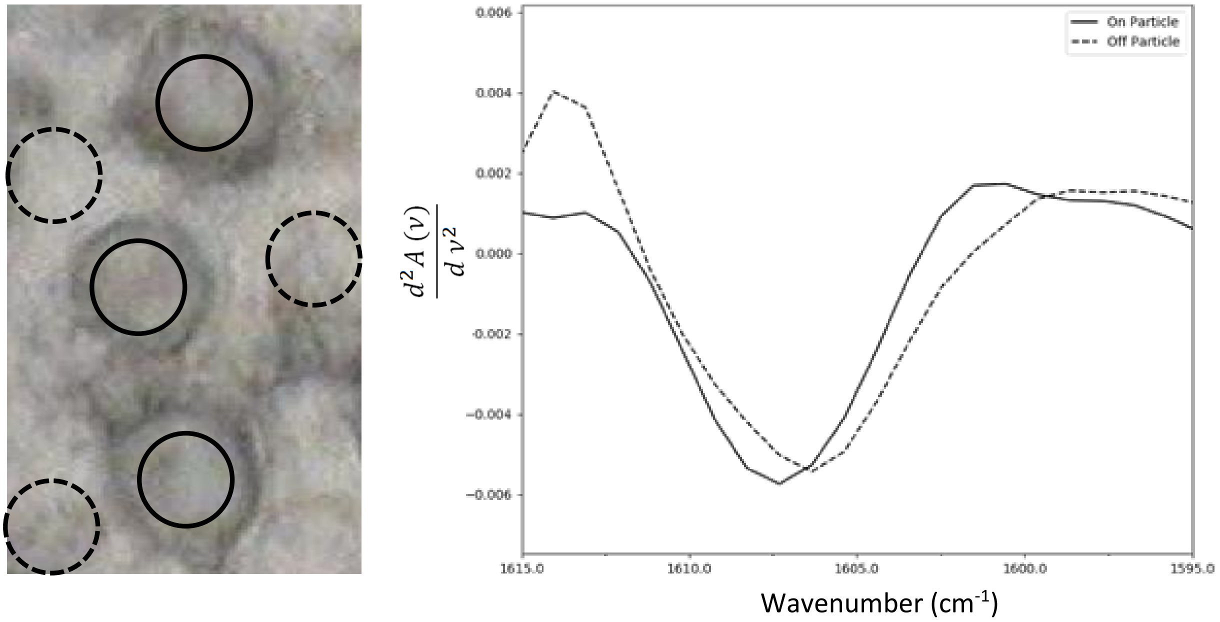

(a)

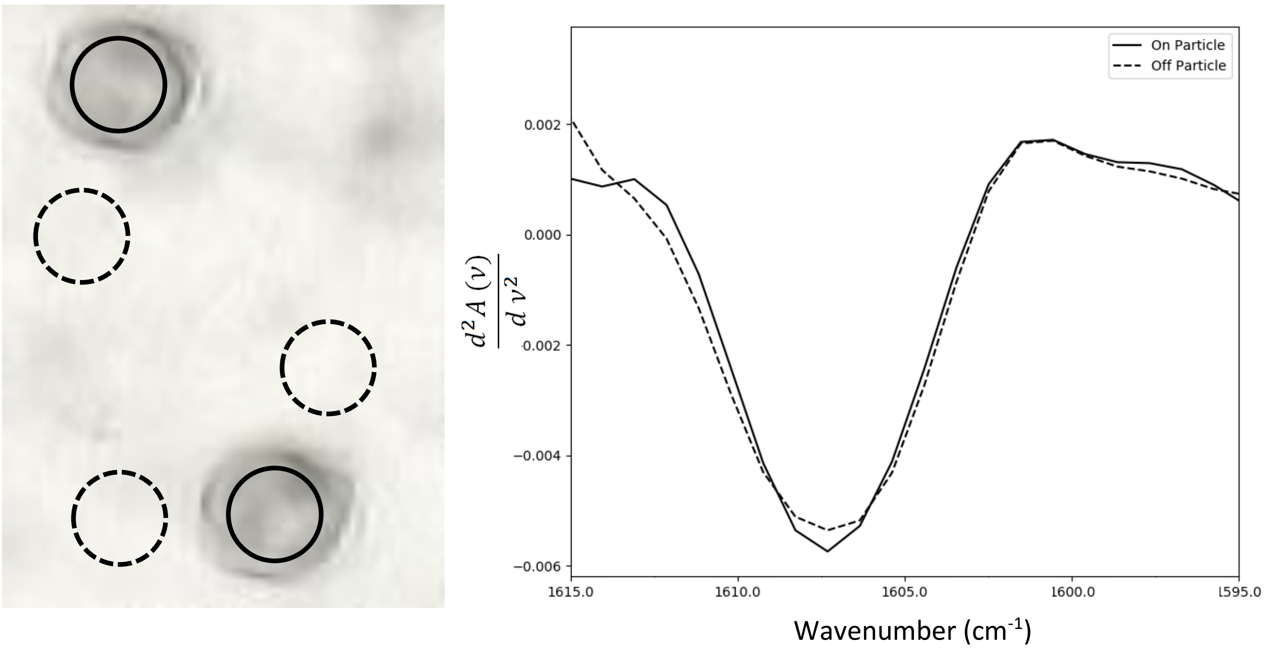

(b)

Supplementary Fig.6. (a) Left: visible image of three 8 µm (diameter) silica beads embedded within a TPO initiated Bis-GMA/TEDMA resin matrix. Full and partial circles correspond to on and off particle positions respectively. Right: second derivative spectra for the corresponding locations in the visible image illustrating a shift in the aromatic wavenumber when moving off particle. (b) Similar analysis undertaken on CQ initiated systems demonstrates no shift in the aromatic wavenumber and is consistent with our findings using spatial oversampling.

Supplementary Table 1. Mean aromatic wavenumber values at on and off particle locations

Photo-initiator	On particle aromatic wavenumber (cm^{-1})	Off particle aromatic wavenumber (cm^{-1})
**CQ**	1608.22 ± 0.81[a]	1608.30 ± 0.72[a]
**TPO**	1608.12 ± 0.40[a]	1606.16 ± 0.56[b]

Supplementary Table 1. Mean aromatic wavenumber values for on and off particle positions within CQ and TPO initiated composite systems obtained via point measurement synchrotron FTIR micro-spectroscopy with a pin-hole optical geometry. Superscripts denote non-significant differences within rows and error bars correspond to the standard deviation.

Supplementary Fig.7: Correlating aromatic strain to polymer chain segment extension

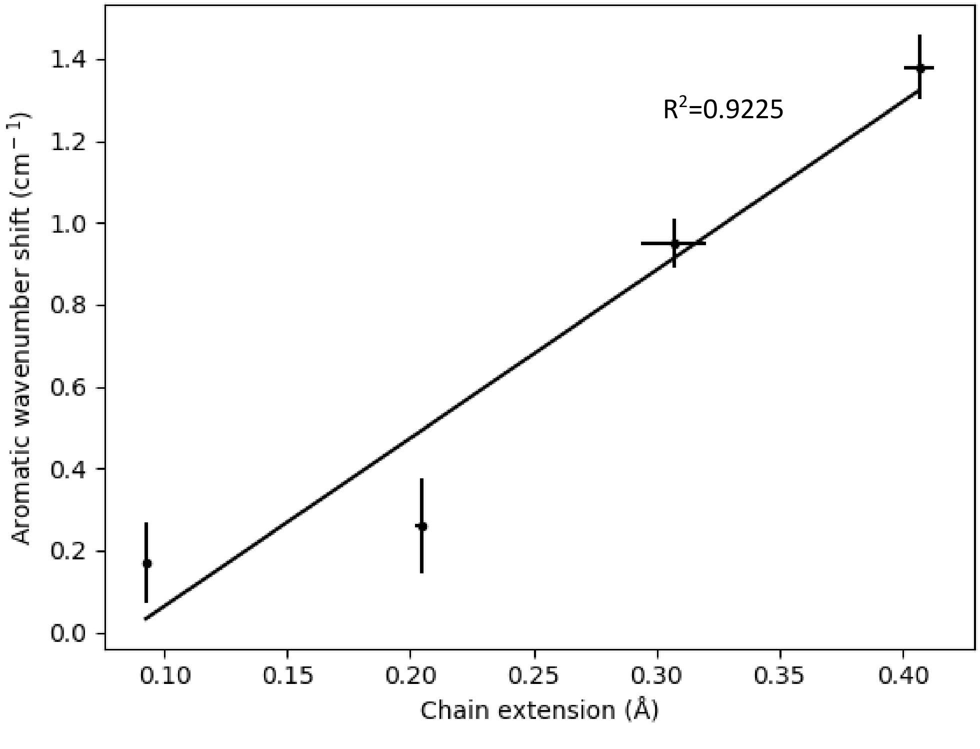

Supplementary Fig.7. The magnitude of the shift in the aromatic absorption band (aromatic strain) as a function of polymer chain segment extension, obtained via X-ray scattering measurements (collected at I22, Diamond Light Source), of identically composed resins under highly similar irradiance conditions to those used in this study. Here, CQ systems polymerized at slower conversion rates are shown by the first two data points, whilst faster systems (TPO) are displayed to the right of the plot. Error bars for aromatic wavenumber and chain extension were obtained via propagation of errors obtained from peak fitting uncertainties.
